# Fate of Gold Nanoparticles in Laser Desorption/Ionization Mass Spectrometry: Toward the Imaging of Individual Nanoparticles

**DOI:** 10.1021/jasms.2c00300

**Published:** 2023-03-14

**Authors:** Vadym Prysiazhnyi, Antonín Bednařík, Michal Žalud, Veronika Hegrová, Jan Neuman, Jan Preisler

**Affiliations:** † Department of Chemistry, Faculty of Science, Masaryk University, 625 00, Brno, Czech Republic; ‡ Nenovision s. r. o., 612 00, Brno, Czech Republic

## Abstract

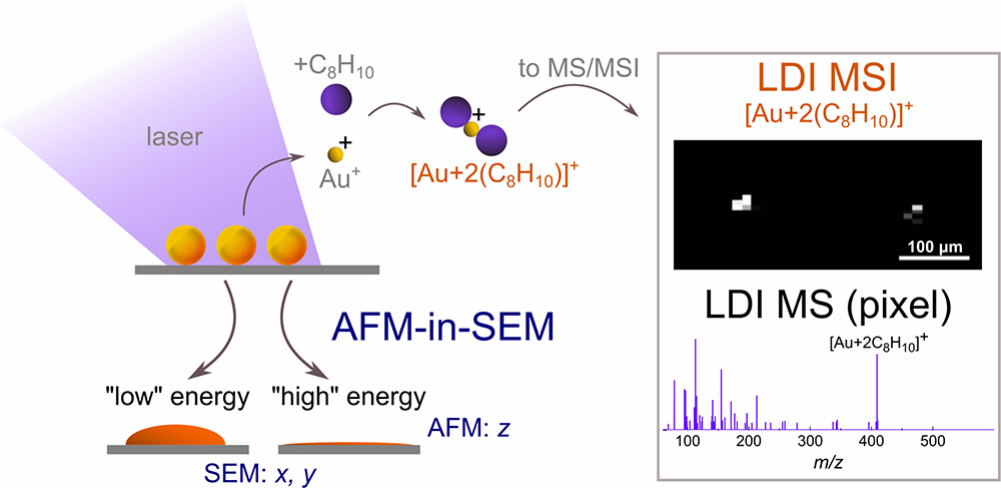

This study focuses on mapping the spatial distribution of Au nanoparticles (NPs) by laser desorption/ionization mass spectrometry imaging (LDI MSI). Laser interaction with NPs and associated phenomena, such as change of shape, melting, migration, and release of Au ions, are explored at the single particle level. Arrays of dried droplets containing low numbers of spatially segregated NPs were reproducibly prepared by automated drop-on-demand piezo-dispensing and analyzed by LDI MSI using an ultrahigh resolution orbital trapping instrument. To enhance the signal from NPs, an in source gas-phase chemical reaction of generated Au ions with xylene was employed. The developed technique allowed the detecting, chemical characterization, and mapping of the spatial distribution of Au NPs; the ion signals were detected from as low as ten 50 nm Au NPs on a pixel. Furthermore, the Au NP melting dynamics under laser irradiation was monitored by correlative atomic force microscopy (AFM) and scanning electron microscopy (SEM). AFM measurements of Au NPs before and after LDI MSI analysis revealed changes in NP shape from a sphere to a half-ellipsoid and total volume reduction of NPs down to 45% of their initial volume.

## Introduction

The utilization of nanomaterials and, in particular, nanoparticles (NPs), in mass spectrometry (MS) has been intensively investigated for decades. Nanomaterials were employed for different purposes: to assist laser ionization and desorption, including selective enrichment of specific molecules, as specific tags for biomolecules,^1−3^ or as a catalytic surface to induce certain chemical or biological processes.^4,5^

NP characterization on surfaces is typically addressed by microscopy techniques, namely scanning or transmission electron microscopy (SEM or TEM),^6,7^ atomic force microscopy (AFM),^8^ or more sophisticated, specially developed methods. But even microscopy techniques will have difficulties resolving NPs in strongly nonhomogeneous (in both chemical composition and topography) biological samples, requiring complex measurement procedures^9^ or the development of new approaches.^10^ For example, the utilization of correlative microscopy, when a few techniques are combined and superposed to get additional information about NP distributions or properties, can be one of the approaches addressing this concern.^11,12^

Mass spectrometry offers both NP detection and characterization, most notably single-particle inductively coupled plasma mass spectrometry (SP ICP MS).^13−16^ In this technique, intact NPs are introduced into plasma producing short, submillisecond signal spikes at mass to charge (*m*/*z*) value(s) specific for the NP element(s). Typically, NPs are introduced to ICP MS from the diluted solution using a nebulizer. An alternative sample introduction technique is laser ablation (LA), where the desorption of NPs occurs, allowing one to count them and map their surface distribution by ICP mass spectrometry imaging (MSI).^14,15,17^ Conventional UV laser ablation results in extensive Au NPs disintegration during the ablation process, which prevents effective NP counting in the SP ICP MS mode. Recently, IR LA ICP MS at the wavelength of 2940 nm has been introduced to minimize damage to NPs during the desorption due to their low absorption in the IR region.^18^

NPs are also common in laser desorption/ionization mass spectrometry (LDI MS). Mostly, the NPs serve here as a matrix in so-called surface-assisted laser desorption/ionization (SALDI) MS,^2,19^ or as selective probes.^20,21^ Also, NPs themselves were the subject of LDI MS investigations. The main advantage of LDI MS over ICP MS is preserving chemical information from the NP surface, which is generally lost in ICP MS. Due to a relatively low laser fluence, LDI allows generating ions of various classes of organic molecules including ones with high biological relevance.^22,23^ This allowed multiplexed NP mapping using LDI MS by tracking various neutral, cationic, or anionic surface-bound ligands (“mass barcodes”) at levels ranging down to pico- or attomoles.^24−26^ Besides metallic ones, graphene nanostructures were also mapped by LDI MS in specific tissue parts.^27^ Direct LDI MS detection of whole relatively small Au clusters (8–29 kDa) modified by different *n*-alkanethiolate ligands^28,29^ or ultrasmall ionized platinum or iron oxide NPs was also reported.^30,31^ The limiting factor of this approach to NP detection is NP size (a few nm at most). Furthermore, in these and other LDI MS studies, NPs were typically used in significant excess, showing the presence of abundant metal ions and adducts.^23,32,33^ We are not aware of any studies that deal with the detection of ions (metals or biomolecules) originating from a few NPs during the surface mapping using LDI MSI experiments.

It is known that NP size affects the number of released metal ions after laser irradiation,^34^ and obviously, a low quantity of released ions from a single NP can be expected. Thus, an essential subject of research arises - the fate of NP under laser irradiation. The physics of the interaction between laser and NPs was investigated in detail due to important applications, such as nanomaterial production in liquids using laser radiation,^35^ laser sintering,^36^ biomedical applications,^37^ and many others.^38^ Both experimental (electron microscopy^39^ or X-ray diffraction^40^) and extensive theoretical investigations^41,42^ were performed. The outcome is that the melting dynamics and temperature exchange are affected by many factors (surrounding environment, nanoparticle material, shape, laser parameters). As a result, the NP heating dynamic (followed by melting/ablation processes) is hard to measure, not to mention predicting it. Authors report a wide range of NP surface temperatures upon laser irradiation ranging from a few tens of degrees,^43^ 800 K,^41^ or even 10^4^ K,^44^ making simple approximations hard even for a rough estimate. However, those temperatures are enough to release metal ions due to surface melting. An exciting report was presented by Levitas and Samani,^45^ where temperature-dependent processes, including melting, were modeled, explaining the mechanism of metal ion release from the NP surface under laser irradiation.

The aim of this work is to tackle whether LDI MS can be used for mapping the spatial distributions of a low amount of NPs, i.e., samples with surface coverage much less than 1% down to a few individual NPs. The following topics will be discussed: (i) the possibility to detect, count, and map a low NP number using LDI MS; (ii) critical limitations and experimental parameters for recording mass spectra of NPs; (iii) physical description of NP behavior during the LDI MS process.

## Experimental Section

### Chemicals and Materials

Ultrapure water was prepared by a Direct-Q 3UV Millipore water purification system (Milli-Q, USA). Methanol (99.8%) was purchased from Merck, USA. Gold NP suspensions with different sizes, shapes, capping agents, and media were purchased from BBI Solutions (U.K.), nanoComposix (USA), and Cytodiagnostics (Canada). The complete list and characteristics of tested NPs can be found in Table S1, Supporting Information.

The substrate for NP deposition was a standard p-doped Si wafer with (111) orientation and <20 Ω·cm resistance (ScienceServices, Germany). After the Si wafer was cut into 3 cm × 2 cm pieces, its surface was cleaned with methanol and carefully wiped with dustless tissue to remove any particles and burrs created by the cutting. The Si wafer substrate was chosen as it is compatible with both SEM and LDI MS.

Thin Au films were deposited using a Quorum Q150T ES sputter coater (Quorum Technologies, U.K.) equipped with a 0.25 mm 2” Au target (99.999%). The prepared film thickness was 6 nm, controlled by a QCM microbalance installed inside a vacuum chamber. The NP suspensions were deposited using a piezoelectric dispensing system equipped with an XYZ stage (Standa, Lithuania). A drop-on-demand MJ-ABP-01-50 DLC piezoelectric dispenser with a 50 μm inner nozzle diameter (MicroFab Inc., USA) was fixed to the *Z*-axis. It was driven by a laboratory-built pulse power supply generating ∼160 pulses/s. Each was a bipolar symmetrically shaped pulse with 30 V amplitude (+30 V for positive, −30 V for negative), 10 μs raise, polarity switch, decay times, and 30 μs dwell time for positive and negative voltages. The volume of the produced single droplets under those conditions was ∼65 pL. The typical size of a single spot on Si wafer was 8–20 μm allowing SEM to image the entire spot and resolve all NPs in it. The number of NPs in each spot was governed by the dilution of the NP suspension. This way, spots containing units to a thousand of NPs were prepared.

**Figure 1:**
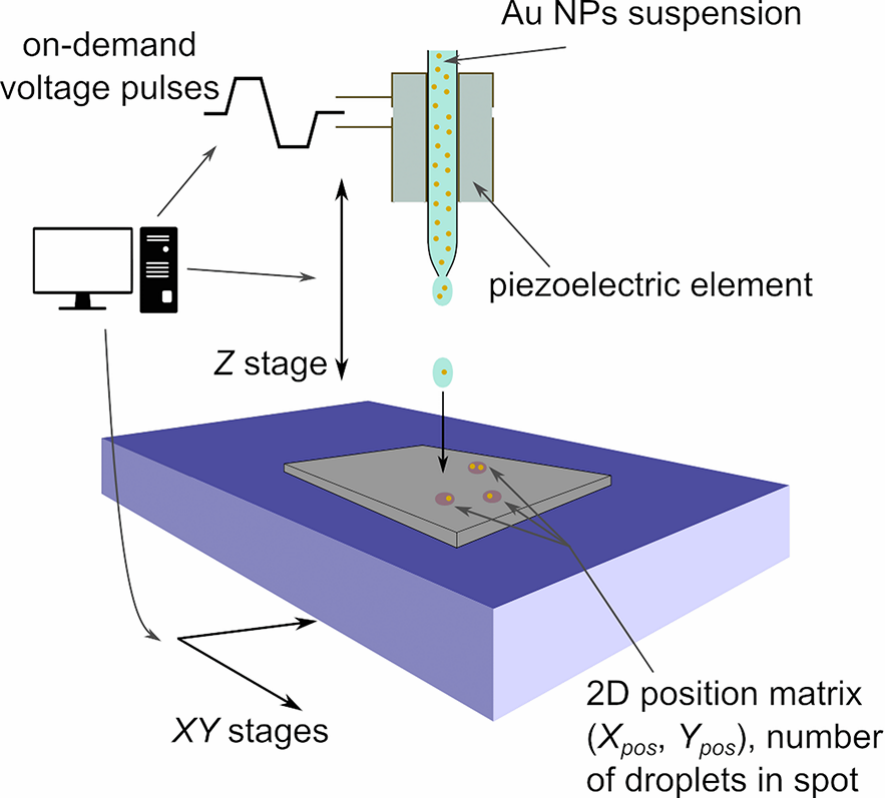
Scheme of a laboratory-built drop-on-demand piezoelectric dispenser for preparation of 2D matrices with low NP numbers.

Figure 1 shows a schema of the experimental setup and on-demand voltage pulse shape.

### SEM

The SEM images were recorded using a Versa 3D electron microscope (FEI, USA). Samples were measured directly without overcoating by a conductive thin film. The acceleration voltage of the primary electron beam was set to 10 kV, and the beam current was 83 pA. Secondary electrons were collected using an Everhart-Thornley detector. The setting of the experimental parameters was adjusted to distinguish NPs based not only on shape, but also on a higher contrast compared to the salt crystals present in the dried droplets. The counting of all NPs was done manually by a thorough inspection of SEM images at magnification 6000–20000×.

### AFM-in-SEM

The correlative AFM-in-SEM imaging was performed using a LiteScope atomic force microscope (NenoVision, Czech Republic) integrated into a MIRA3 XMU electron microscope (Tescan, Czech Republic). This setup helped identify the regions of interest and quickly navigate the tip thanks to the SEM wide-field. Here, an Akiyama self-sensing probe (NV-A-Probe-10, Nanosensors, Switzerland) with a visible apex was used for topography measurement. It employs a tuning fork to measure the surface topography in the frequency-modulated tapping mode. The images were acquired in the closed-loop with 1500 × 1500 points, getting material contrast from a secondary electron detector, with an acceleration voltage of primary electron beam of 10 kV and beam current 83 pA.

### LDI MS

The samples with deposited Au NPs were analyzed inside a dual subatmospheric MALDI/ESI ion source (SubAP/MALDI (ng), MassTech Inc., USA) connected through ion funnels to an Orbitrap Q Exactive Plus mass spectrometer (Thermo Fisher Scientific Inc., Germany). The LDI target is placed opposite the entrance orifice into the high-vacuum part of the mass spectrometer, while the ESI capillary enters the ion-funnel region perpendicularly. The ion source was equipped with a 355 nm Nd:YAG laser with low energy (<1 μJ/pulse) at 1 kHz frequency. The sample imaging was done in continuous raster mode (CRM); i.e., the sample plate was moving at a constant speed, while the laser was shooting continuously. The movement parameters (horizontal speed, vertical step between the scanning lines) determine the pixel size. Though final measurements were done with a 15 μm × 7 μm pixel size, the pixel size varied during the initial optimization process. To enhance the ion signal of Au-containing ions, xylene vapor was introduced to the ion source (a small beaker with 5 mL of xylene was placed in a confined space near the ESI capillary inlet). The mass spectra were recorded in the *m*/*z* range from 50 to 750 Da with 35000 resolution (at *m*/*z* 200 Da) and 239 ms injection time (IT). The respective sample table horizontal speed was 3.44 mm/min, resulting in a single-pixel acquisition time of 261 ms (increased by ∼22 ms for ion injection from C-trap to the orbital trap). The acquisitions were performed in leveraged mode with the C-trap prescan turned off and in fixed AGC mode. Therefore, IT was kept at 239 ms independently on the input ion number. Transient signal measurement inside the orbital trap was executed simultaneously with ion injection from the next pixel to C-trap, minimizing the ion losses.

The utilized laser spot did not have a flat-top profile, and its size depended on the laser energy (for the laser energy of 0.31 μJ/pulse, the laser spot was 14.5 μm in horizontal diameter). The positioning system tolerance was ±1 μm. Within the used laser energy range, 7 μm distance between laser beam lines on the target ensured complete sample surface irradiation and was used in all LDI MSI experiments. The selection of appropriate imaging parameters for MSI has been discussed before.^46,47^

Irradiating the whole surface is not substantial in typical MALDI MSI experiments (the pixel size can be higher than the actual laser spot), but it was a crucial factor in NP LDI MSI experiments to ensure hitting every NP in the sample. The details on the spot size and the width of the scan line depending on the laser energy in the used experimental setup can be found in Figure S2b, Supporting Information.

The laser energy was measured using a PM160 optical power meter (Thorlabs, USA) by placing the sensing unit after the last focusing lens before the sample plate. The measurements were done in the complete dark. The background signal was averaged at least for 20 s (200 measurement points), followed by at least 40 s measurement with the laser turned on (400 measurement points). The power signal was later recalculated into energy per pulse. The signal RSD was less than 1.2% of the measured value.

## Results and Discussion

### LDI Mass Spectra from Au Nanostructures: From Nanolayer to a Few NPs

**Figure 2:**
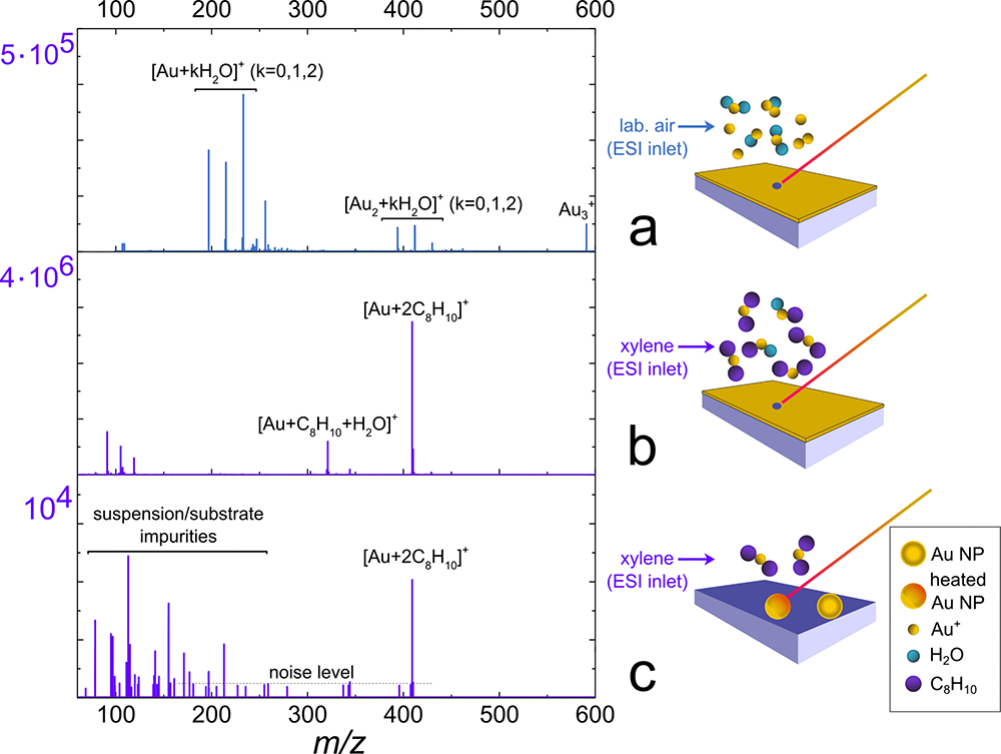
LDI mass spectra generated from 6 nm thick Au film with (a) ambient air influx and (b) xylene vapor influx; (c) from a few 50 nm PEG-coated Au NPs with xylene vapor influx.

First LDI MS experiments were done with 6 nm Au film prepared using the magnetron-sputtering technique to ensure an abundance of generated Au ions. An influx of laboratory air into a subAP MALDI/ESI source resulted in a generation of a few types of ions: charged gold clusters (Au_*n*_^+^: Au^+^ at *m*/*z* 196.966, Au_2_^+^ at *m*/*z* 393.933, Au_3_^+^ at *m*/*z* 590.899) and adducts of gold and water molecules ([Au + H_2_O]^+^ at *m*/*z* 214.977, [Au + 2(H_2_O)]^+^ at *m*/*z* 232.987, [Au_2_ + H_2_O]^+^ at *m*/*z* 411.943, and so on); see Figure 2a. The introduction of xylene vapors resulted in the preferential formation of two adducts: [Au + 2(C_8_H_10_)]^+^ at *m*/*z* 409.123 and [Au + C_8_H_10_ + H_2_O]^+^ at *m*/*z* 321.055 (Figure 2b). It was important as the released Au ions distributed initially among multiple ion types were consumed and formed only the two preferential adducts. The signal intensity of [Au + 2(C_8_H_10_)]^+^ ion reached 2.7 × 10^6^ a.u., more than an order of magnitude higher compared to the Au^+^ signal with laboratory air influx (2.2 × 10^5^ a.u.). Adding vapor of other volatiles can have similar effects on Au ion signal enhancement (benzene, acetonitrile) or can lead to an overall signal drop (alcohols, aldehydes).^48^ Still, the signal enhancement by the selective gas-phase reaction was most pronounced for xylene. Furthermore, xylene evaporates slowly, and thus a single beaker with 5 mL of the solvent could be used for 3-day experiments (more than 60 h of measurement) with no memory effect. This allowed achieving the sensitivity necessary for detecting low counts of Au NPs even during long LDI MSI experiments. For example, the mass spectrum in Figure 2c originates from a pixel containing a few 50 nm PEG-coated Au NPs recorded from a dried 65 pL droplet spot containing ∼50 NPs (a droplet was typically displayed on 2 × 2–3 × 4 pixels in the MS map; the NP number in the spot was estimated from the concentration provided by the NP suspension manufacturer). The absolute signal intensity drop of the Au/xylene adduct ion is due to a simple decrease in gold ion quantity. The presented impurities in Figure 2c at lower *m*/*z* originate from the NP suspension.

### Selection of Au NP Suspension

**Figure 3:**
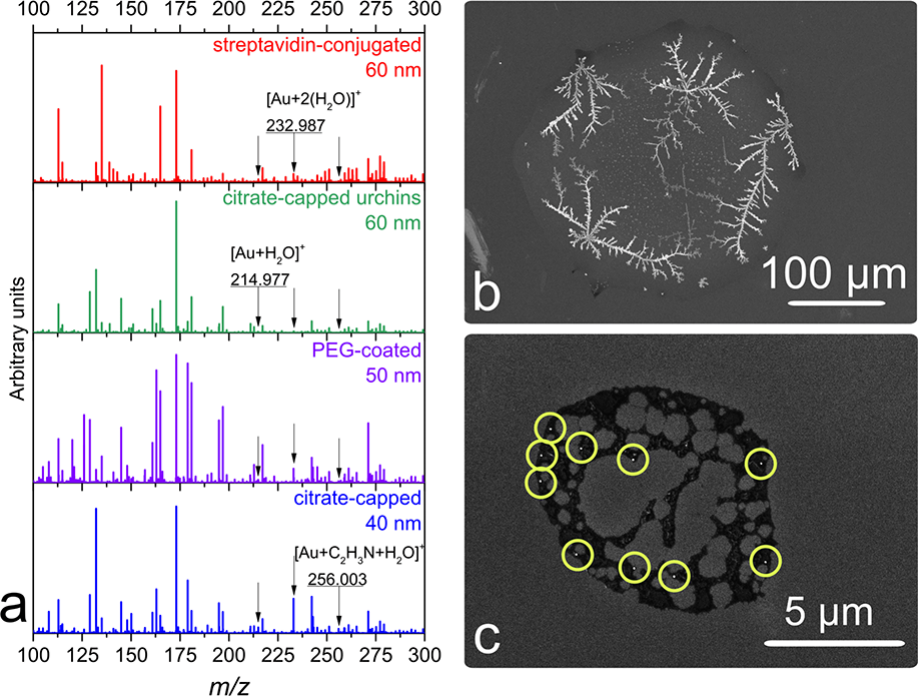
(a) LDI mass spectra from 0.5 μL droplets of selected Au NP stock suspensions. SEM images of dried droplets from (b) 0.5 μL 60 nm streptavidin-conjugated Au NP suspension and (c) 65 pL 50 nm PEG-coated Au NP suspension (yellow circles highlight the position of NPs), showing the deposition of a few Au NPs achieved with a diluted NP suspension.

The samples with low Au NP numbers were deposited as microscopic droplets of diluted NP suspensions using an on-demand piezoelectric dispenser. These systems were used for similar purposes to deliver single analyte counts.^49,50^ The requirements for suspension were (1) simple LDI mass spectrum and (2) the ability to resolve each deposited NP using SEM. From the mass spectrometry point-of-view, Au-containing ions can be observed from each examined suspension even in the background coming from additives. The MS spectra were recorded from 0.5 μL as-received suspensions with high NP counts and high concentrations of additives. See the example in Figure 3a, where averaged spectra over several hundred pixels obtained from a few scan lines across the dried spots are shown. However, to meet the second requirement of resolving each NP in SEM, suspensions with high additive content cannot be used, as larger crystals and tree-like structures were observed after droplet drying (Figure 3b). Even 1000× diluted citrate-capped bare NPs formed small (size close to the NP diameter) and large (over 500 μm) crystals. NPs are harder to resolve when they are located in the vicinity of nonconductive crystals, which give false-positive contrast. One of such examples is presented in Figure S1a, Supporting Information. Among the tested NP suspensions, a clear identification of Au NP from “background interferences” during SEM was possible only in the case of the suspension of PEG-coated Au NPs stabilized in citrate. The diluted suspension of these NPs allowed reliable counting of individual particles using SEM as no or only a small amount of interfering salt crystals were present in the sample. Figure 3c shows an example of a desired dried droplet spot with a diameter of 8 μm prepared by piezo-depositing a single 65 pL droplet of properly diluted suspension. Depending on substrate preparation conditions, 65 pL droplets deposited individually on Si wafer produced spots with diameters ranging from 6 to 20 μm. The secondary electron mode was used to differentiate Au NPs, inorganic additives, and Si substrate.^51^ The bright white dots are 50 nm Au NPs, the black color represents a thin layer of additives in a suspension, and the gray color is the Si substrate.

### Design and Preparation of Sample

A piezoelectric dispenser was used for reproducible preparation of arrays of 4 × 4 dried-droplet spots with spacing 250 μm. Due to the pixel size 11.2 to 3.4 nm at used SEM magnifications 6000–20000×, all NPs can be resolved in the resulting 6144 × 4415 SEM image.

As expected, the actual number of NPs in the individual spots varied, and its dispersion increased for spots containing fewer NPs. The RSD of the NP number in spots with 1000, 500, 50, and units of NPs was 5%, 10%, 35%, and up to 800%, respectively. For example, within a 10 × 5 matrix of spots with average 2.4 NPs/spot, a spot containing 19 NPs was measured. Such differences were typically a result of NP aggregation or Poisson distribution.

### MSI of Areas with Low NP Numbers and Characterization of the Irradiated Spots

**Figure 4:**
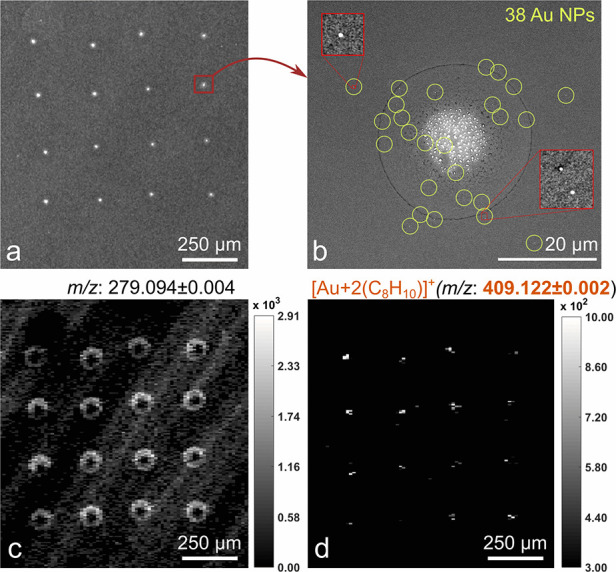
SEM images of (a) a spot array at low magnification; (b) a single spot (yellow circles highlight positions of NPs); MS maps (c) showing organic impurities concentrated at the spot edges at *m*/*z* 279.094 ± 0.002; (d) of [Au+2(C_8_H_10_)]^+^ adduct at *m*/*z* 409.122 ± 0.002.

Nevertheless, SEM allowed counting the number of deposited Au NPs on the given Si substrate. The SEM image of one of the spot arrays containing ∼50 NPs per spot, as calculated from the NP suspension manufacturer data, is shown in Figure 4a. Note that the spots were not ideally aligned due to droplet drying. The observed irregularities helped confirm the alignment of MSI data with SEM images. It was verified by SEM that the number of 50 nm PEG-coated Au NPs ranged between 15 and 60 NPs in each spot (Figure 4b; an additional SEM image is provided in Figure S3, Supporting Information). The NPs had a perfect round shape and higher brightness compared to salt crystals, which allowed their discrimination. The exact NP counts from two of such spot arrays are shown in Table 1, column “NP number (SEM)”. The MSI (used laser energy was 0.31 μJ/pulse) map in Figure 4c illustrates the spatial distribution of one of the background organic ions (*m*/*z* = 279.094) located at the outer droplet edges.

**Table 1:** Average Number of NPs in a Single MSI Data Pixel Derived from Two 4 × 4 Spot Arrays^a^

	Spot array #1			Spot array #2		
spot	NP number (SEM)	nonzero data pixels (MS signal)	NP/pixel (MSI)	NP number (SEM)	nonzero data pixels (MS signal)	NP/pixel (MSI)
1	31	3	10.3	39	6	6.5
2	48	6	8.0	39	5	7.8
3	59	8	7.4	64	6	10.7
4	33	4	8.3	44	3	14.7
5	16	2	8.0	39	5	7.8
6	20	2	10.0	40	7	5.7
7	32	2	16.0	65	8	8.1
8	25	3	8.3	57	4	14.3
9	40	2	20.0	19	4	4.8
10	22	1	22.0	25	4	6.3
11	36	4	9.0	56	3	18.7
12	32	4	8.0	54	4	13.5
13	26	2	13.0	21	2	10.5
14	24	3	8.0	22	2	11.0
15	31	3	10.3	31	4	7.8
16	39	4	9.8	51	5	10.2
Average	32 ± 11		11 ± 4	42 ± 15		10 ± 4
Average NP/pixel for both spot arrays				11 ± 4		

a Number of NPs was ∼50 NP per spot as calculated from the concentration provided by the NP suspension manufacturer. The average signal value per pixel was 560 ± 180 a.u.

Most importantly, MSI of [Au+2(C_8_H_10_)]^+^ ion at *m*/*z* 409.122 (Figure 4d) points precisely to the Au NP positions in the centers of the spots. Thanks to the negative mass defect of the Au^+^ ion (*m*/*z* = 196.966), ppm mass accuracy, and high resolving power of the orbital trap, the probability of interference from organic ions is minimal. Only very few pixels in LDI MSI maps outside the borders of dried droplets with the signal at *m*/*z* 409.122 ± 0.002 were present, meaning the chemical noise at this *m*/*z* region was negligible. Note that the measured ions are not coming from a single NP at this stage. Single NPs deposited on Si substrates did not yield measurable signal underexamined measurement parameters.

The next step was correlating pixels containing Au adduct ion signals with the physical location of the NPs obtained by SEM. Though the pixels containing signal of [Au + 2(C_8_H_10_)]^+^ were present almost exclusively in the center of the dried spots, the position of these pixels was hard to correlate with actual NP positions within the dried spots. There might be several reasons for this; first is the low MSI resolution limited by the laser spot size, and second is the NP delocalization during LDI scanning, which is shown later. Table 1 shows estimations of NP numbers presented as numbers of nonzero pixels (i.e., pixels with Au-related signals above noise level). It was decided to work rather with the NP/pixel values than with the signal/NP ones, as the signal of single NP would be under the instrumental noise level. Averaging the signal intensity of nonzero pixels (Figure 4d) resulted in 560 ± 180 a.u. signal intensity, which is more than twice as high as the instrumental noise level under given measurement conditions (260 ± 20 a.u.). In rare cases (less than 2% of pixels containing Au-related ions), the presence of NP aggregates resulted in pixels with a signal over 10000 a.u. These were not included in the average as we wanted to measure only individual NPs. On average, 11 ± 4 of 50 nm NPs were responsible for signal generation in the nonzero pixels of the MS image, as is summarized in Table 1. At this moment, this is a rough estimate of the LDI MSI detection limit under current experimental conditions.

**Figure 5:**
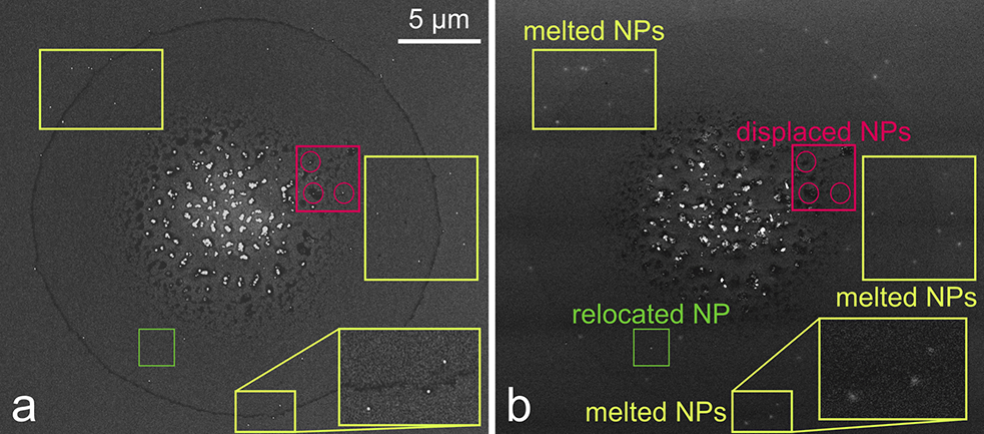
Effect of laser irradiation at 0.41 μJ/pulse during MSI run and its impact on 50 nm PEG-coated Au NPs showing melted and relocated NPs on SEM image of a spot (a) before and (b) after laser irradiation.

Then, the samples irradiated by laser were remeasured using SEM. Figure 5 shows a single spot before and after laser irradiation to demonstrate the outcomes of these investigations qualitatively. With specific microscope settings (increasing contrast to the maximum and brightness to a minimum), a white “halo” appears at the original positions of Au NPs on the surface after the laser irradiation. Note that most NPs stayed in their original places (yellow areas), while some were relocated to new positions (three NPs from the red area disappeared, while a single NP appeared in the green area). As Au NPs were not bonded to the substrate (through functional groups or other means), one can expect that laser irradiation could lead to a change in their position. Based on the SEM images measured before and after laser irradiation from the same spot array, 402 Au NPs were identified. From this total count, 313 (78%) NPs were melted on the same spot, while 89 (22%) NPs were relocated as a result of the laser irradiation.

### Correlative AFM-in-SEM Imaging: What Happened to NPs during LDI MS?

SEM images suggest that NP melts similarly to a ball of chocolate exposed to heat. However, SEM gives only a flat picture, in which contrast depends on the chemical composition of the topmost layer. The incorporation of height determined by AFM would help understand the phenomena. This is crucial in the complex system consisting of Si substrate with both Au NPs and additives deposited on the same spot. SEM is well-known for giving actual lateral dimensions, while AFM topography is accurate on the *Z*-scale. 100 nm PEG-coated Au NPs were utilized for these experiments as a larger NP size allows one to measure smaller changes in the NP morphology. Three 65 pL droplets were deposited on each spot within an array of 7 × 15 spots with 300 μm spacing in both *X* and *Y* directions. Each spot contained around 100 Au NPs, as verified by SEM. The prepared array was scanned by a laser with the horizontal scan speed kept the same as in the case above. Vertical line spacing was intentionally increased to 30 μm, leaving some areas without laser irradiation after the MSI experiment. For every 20 scan lines (each 600 μm), the value of laser energy was increased from the initial value of 0.1 μJ/pulse to 0.48 μJ/pulse. Thus, strips with partially irradiated spots containing both melted and intact NPs were available. This was important to collect data from enough NPs for statistical evaluation, and for easy spot location (for practical reasons, nonablated spots are simple to track due to contrast obtained from NP suspension additives).

**Figure 6:**
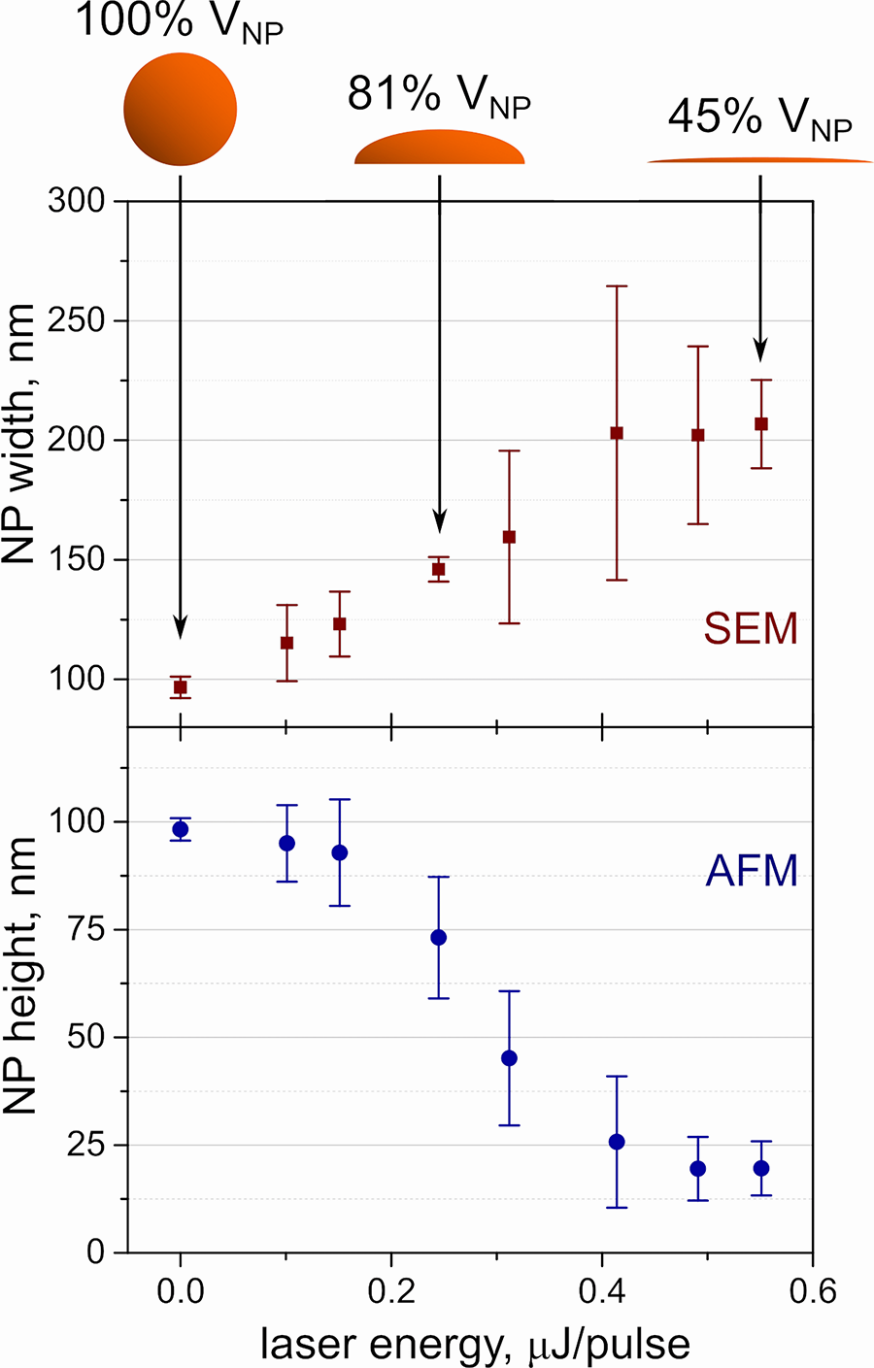
Experimentally determined NP width (from SEM) and height (from AFM) as a function of laser energy. Error bars represent standard deviation from measuring 10 individual NPs.

Figure 6 shows the measured width and height of NPs, i.e., the diameter of the droplet determined by SEM and AFM, respectively. Each point is an average from 10 NPs irradiated by a laser pulse with a specific laser energy level. The deposited, intact NPs were 98.2 ± 2.6 nm in height (measured from horizontal profiles passing through the NP center from AFM data) and 96.6 ± 4.5 nm in width (as a horizontal brightness profile passing through the NP center from SEM data). These values are in agreement with the diameter specified by the producer of the Au NPs. The NP changes induced by laser irradiation support the initial perception of melting chocolate balls exposed to heat: with increasing laser energy per pulse, the NPs become lower while getting wider in the lateral dimensions. At the higher values of laser energy, the spherical particle morphs into a pile with 20 ± 6 nm height (AFM data) and 209 ± 19 nm width (SEM data). To qualitatively evaluate the loss of NP mass, a simple model in which NP irradiated by laser transforms from a sphere to a half-ellipsoid is proposed. In this case, irradiation of 0.25 μJ/pulse reduces its volume to 81%, while irradiation of 0.55 μJ/pulse reduces its volume to 45% of the initial NP volume obtained from AFM-in-SEM experiments.

**Figure 7:**
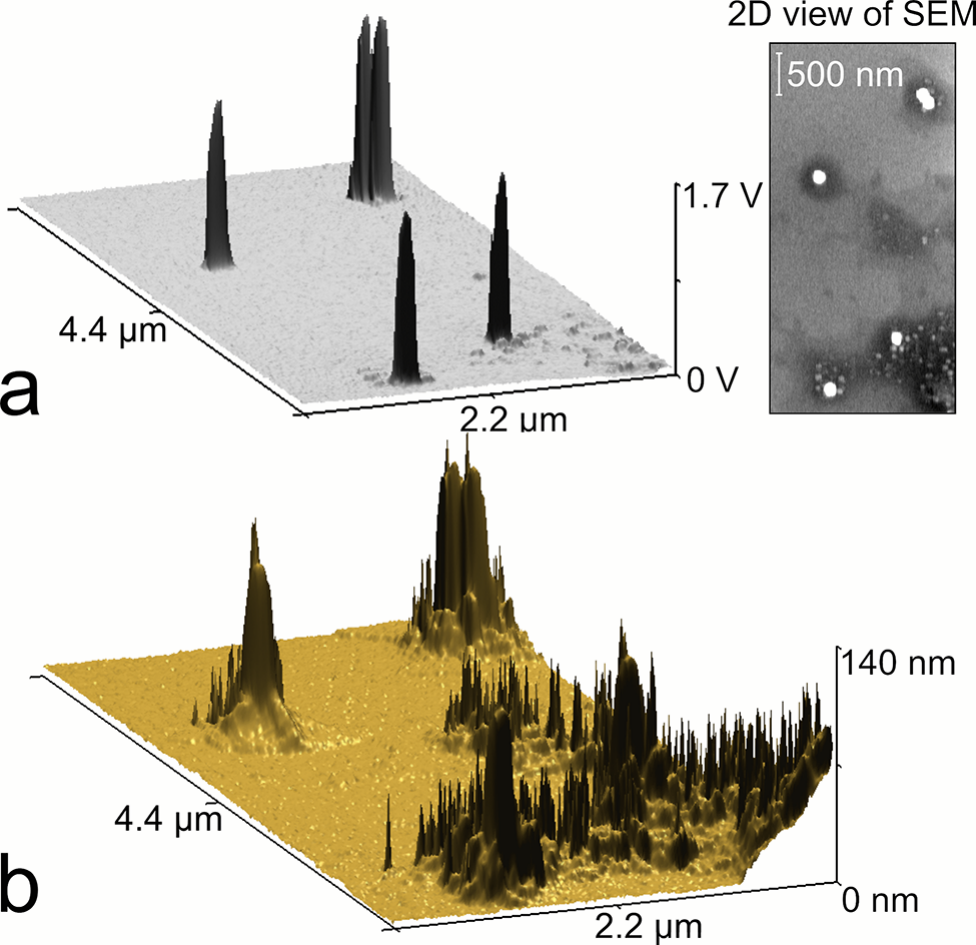
Correlative SEM-in-AFM imaging. 3D topography of area containing five 100 nm PEG-coated Au NPs measured using (a) SEM and (b) AFM.

Additionally, using AFM-in-SEM revealed that Au NPs were not laying on the Si substrate but were partially embedded in solid residue from the diluted suspension. Electron microscopy alone could not provide this information during counting NPs, as is highlighted in the images of the same five Au NPs using SEM and AFM in Figure 7a and 7b, respectively. A typical 2D view of an electron microscopy image provides material contrast, but even if plotted in 3D, it does not show actual topography, though it can be anticipated based on the color contrast from a 2D view. On the other hand, AFM clearly shows that each NP is embedded in a residue, substantially increasing the contact area with the substrate. The observed differences in the number of additives surrounding each NP most probably result in different NP adhesion to the substrate; thus, a relocation of some NPs shown in Figure 5 can be anticipated. If this hypothesis is correct, NP embedding decreases the number of relocated NPs during laser irradiation. More details about laser ablation of these residues are demonstrated in Figure S4, Supporting Information.

## Conclusions

This article shows the capability of the LDI MSI technique to display spatial distributions of tens of NPs, which has not been reported before. The research was focused on the fundamental investigations starting from sample preparation, evaluating effects of laser fluence in LDI of individual NPs, and detailed description of laser-NP interaction (ablated ions, morphological changes).

The drop-on-demand piezoelectric dispenser presents an effective tool for preparing 2D arrays of spots with sizes down to tens of μm containing low NP numbers within a specific range. The exact number of deposited NPs depends on many factors: aging of the stock NP suspension, presence of NP aggregates, pipetting errors during dilution steps, adsorption of NPs on walls during sample preparation, and statistical probability of NPs entering the piezodispenser orifice. Thus, the accurate number of deposited NPs must be determined using SEM in each spot. The LDI MS technique was found to be sensitive enough to detect low Au NP numbers as well as the presence of organic additives of NP suspensions. Compared to classical LDI/MALDI MSI, where pixel size can be significantly larger than the laser spot size, the measurement conditions must be carefully optimized when the need to irradiate the whole sample surface, i.e., all NPs, arises. This requires knowledge of the laser spot profile and a detailed understanding of the MS scanning mode.

In order to increase sensitivity enabling the detection of low Au NP numbers, vapors of xylene were introduced via the ESI capillary of the dual MALDI/ESI source. Thus, most of the multiple signals originating from various gas phase reactions with water and other volatiles present in laboratory air were converted into an intense peak of [Au + 2(C_8_H_10_)]^+^ adduct ion with the summed intensity of the Au-containing ions. At the moment, the detection limit of Au NPs was estimated as low as 11 ± 4 NPs deposited on the Si substrate within a 15 μm × 7 μm MSI pixel. The development of mass analyzers with higher sensitivity or further optimization of experiments will lead to the ultimate goal of detecting individual single NPs on the surface using the LDI MS technique. Recently introduced Orbitrap mass analyzers boast about an order of magnitude higher sensitivity compared to the instrument used in this work and might approach the detection of a single NP.

The SEM and LDI MSI multimodal imaging approach faces numerous challenges. A proper NP suspension compatible with both techniques has to be selected, especially with regard to the nature of stabilization agents. Crystallization of these additives in dried droplets can hinder the SEM measurement of even excessively diluted NPs solutions. For this reason, of all tested NPs suspensions, only one model NP suspension compatible with both techniques was found: PEG-coated Au NPs stabilized in an aqueous citrate solution. It does not mean that the LDI MS approach will not work with suspensions where the amount and variety of additives are high, but another independent method for NP counting would have to be developed.

Correlative AFM-in-SEM allowed the changes in the NP geometry (height using AFM and lateral width using SEM) to be directly tracked. The observed embedding of NPs in the residue of additives can be a reason for the witnessed relocation of NPs. About 22% of NPs moved to new positions for used experimental conditions, while 78% stayed in their original place after laser irradiation. While the energy dose that a single NP receives can be calculated as the number of pulses multiplied by NP area and laser fluence, we are unaware of any similar work going into such details experimentally. With increasing laser energy, the shape of NP was gradually changing from spherical to “semielliptic”. This also changes plasmonic properties, and thus there might be a threshold of laser energy after which the Au signal does not increase further. Such behavior of individual NPs differs from the published phenomena concerning large amounts of neighboring NPs, which can lead to particle joining and formation of larger particles.^52^ In the future, the unique combination of AFM-in-SEM directly visualizing NP shape changes can help to develop better models for understanding NP melting and interaction with lasers.

## Supporting Information Available

Table providing information about tested NPs in this work, examples of single NP resolution using SEM, the result of the difference in pixel size and irradiated spot size, details on laser spot size and scan line width, and AFM topography of laser irradiated spot (PDF)
