# Supplementary material for: Fate of Gold Nanoparticles in Laser Desorption/Ionization Mass Spectrometry: Toward the Imaging of Individual Nanoparticles
Source: J Am Soc Mass Spectrom. 2023 Mar 14;34(4):570–8. doi: 10.1021/jasms.2c00300 (PMC10080673; doi:10.1021/jasms.2c00300)
Supplement: Supplementary file 1 — js2c00300_si_001.pdf [file js2c00300_si_001.pdf]

Supporting Information

Fate of gold nanoparticles in laser desorption/ionization mass spectrometry: towards the imaging of individual nanoparticles

Vadym Prysiazhnyi[1], Antonín Bednařík[1], Michal Žalud[1], Veronika Hegrová[2], Jan Neuman[2], Jan Preisler[1*]

[1]Department of Chemistry, Faculty of Science, Masaryk University, 625 00 Brno, Czech Republic

[2]Nenovision s. r. o., 612 00 Brno, Czech Republic

Table of Contents

Table S1. Au NPs used in this work and their principal properties S-2

Detailed look on a single spot, negative effect of suspension additives and incomplete sample irradiation S-3

Laser spot shape and dependence of laser spot height (Y) on the laser energy S-4

Detailed look on a single spot prepared by the piezoelectric dispenser showing individual standing NPs and the differentiation between NPs and salt additives S-5

AFM topography of a laser-irradiated spot and re-ablation of suspension additives S-6

Table S1. Au NPs used in this work and their principal properties

**NP size, nm**	**Producer, catalog number**	**Nominal NP concentration, NP/mL**	**Capping agent**	**Shape**	**Media**
40±3	BBI Solutions, EM.GC40/7	$9 \cdot 10^{10}$	citrate	spherical	2 mM sodium citrate
50±2	nanoComposix, AUXU50-1M	$9 \cdot 10^{10}$	PEG	spherical, ultra-uniform	2 mM sodium citrate
102±4	nanoComposix, AUXU100-1M	$4.9 \cdot 10^{9}$	PEG	spherical, ultra-uniform	2 mM sodium citrate
60±6	Cytodiagnostics, GU-60-20	$1.96 \cdot 10^{10}$	citrate	flower-like urchins	0.1mM PBS
60±7	Cytodiagnostics, AC-60-04-05	$5.88 \cdot 10^{10}$	streptavidin	spherical	1xPBS (pH 7.4), 20% glycerol (v/v), 1% BSA

Detailed look on a single spot, negative effect of suspension additives and incomplete sample irradiation

The deposited spot is about 15 μm in diameter and contains 60 nm citrate-capped Au NPs (Fig. S1a). Note that even for citrate-capped Au NPs in water, the non-conductive crystals give false-positive white contrast, and they can be either identified as NPs (in case salt crystals have a size similar to that of NPs) or can cover Au NPs, thus reducing the NP count.

Fig. S1b shows laser irradiating the surface with 20 μm spacing between lines, intentionally demonstrating the effect of incomplete sample irradiation. Here, 8.7 μm-wide areas in between lines were not irradiated by the laser.

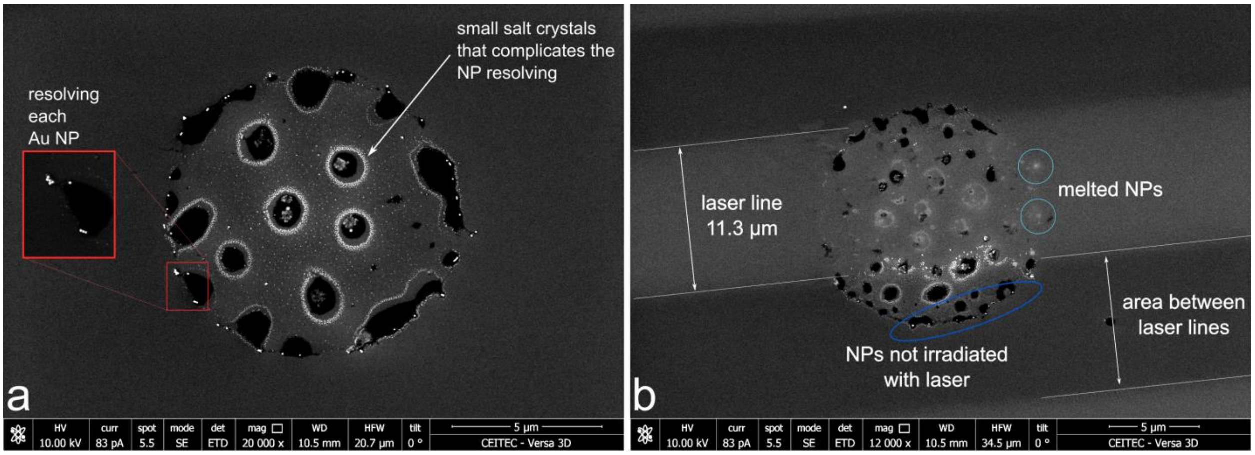

Fig. S1. SEM images of (a) a dried spot prepared using the piezo-dispenser with several 60 nm citrate-stabilized Au NPs, (b) another spot scanned with 0.51 μJ/pulse laser energy with 20 μm distance between the lines showing irradiated and non-irradiated NPs

Laser spot shape and dependence of laser spot height (*Y*) on the laser energy

Fig. S2a shows a laser spot ablated in 6 nm Au film under conditions similar to the MSI run (400 shots at 0.41 μJ/pulse energy) imaged by SEM. Note that the laser spot does not have a flat-top profile; the center of the spot with the vertical (*Y*-axis) size of 7 μm is surrounded by ~1.8 μm ring with leftovers of Au film.

The irradiated Si substrate was used to find the average thickness of the laser scan line, as contrast lines were visible after irradiation. Fig. S2b shows experimentally observed traces (lighter areas) after laser line scans depending on the laser energy values. The distance between the centers of the scan lines was set to 20 μm.

Fig. S2c recapitulates the processed data (averaged from at least 10 positions of the irradiated traces on two samples). The trace width increased from 4.0 μm at laser energy 0.1 μJ/pulse to 9.7 μm at 0.41 μJ/pulse.

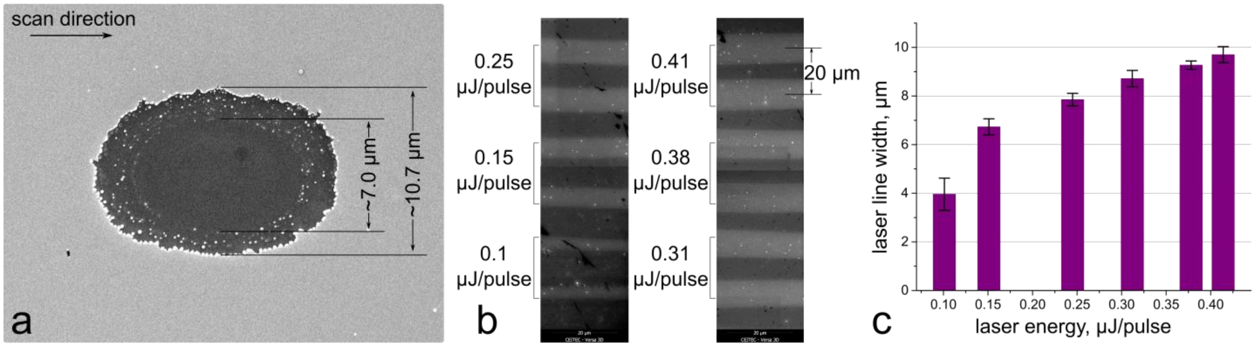

Fig. S2. (a) SEM image of a spot ablated in 6 nm Au film by 400 laser pulses at 0.41 μJ/pulse energy; (b) combined SEM images of the traces on Si substrate produced at different laser energy levels; and (c) the dependence of the trace width on laser energy

Detailed look on a single spot prepared by the piezoelectric dispenser showing individual standing NPs and the differentiation between NPs and salt additives

To demonstrate the possibility of distinguishing between the NPs and impurities, another example of the 'unprocessed' SEM image is represented below. Here, based on shape and contrast differences, the detection and counting of NPs are unambiguous.

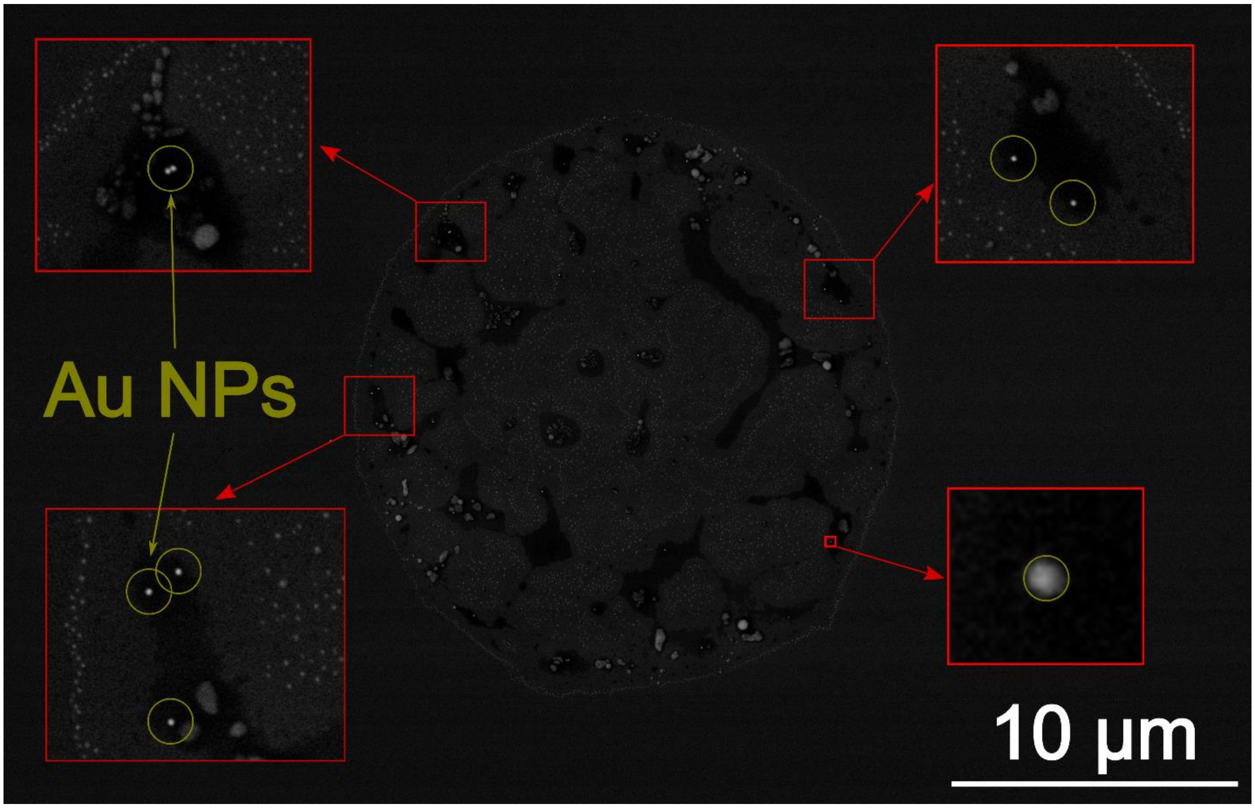

Fig. S3. Unprocessed SEM image of dried 65 pL droplet spot with 50 nm Au NPs prepared using piezoelectric dispenser showing a clear distinction between the NPs and salt additions.

AFM topography of a laser-irradiated spot and re-ablation of suspension additives

The dried droplet spot in Figure S4 was irradiated by laser at 0.15 μJ/pulse with a 10 μm distance between the centers of the laser lines. AFM shows that the laser ablates most of the residue from the Si surface. The line profiles extracted perpendicularly to the laser scanning also suggest that either spreading or re-deposition of residue appears at the edge of the scan lines. The laser interaction with the residue adds one more unknown variable to the energy balance and heat exchange between laser radiation and NP.

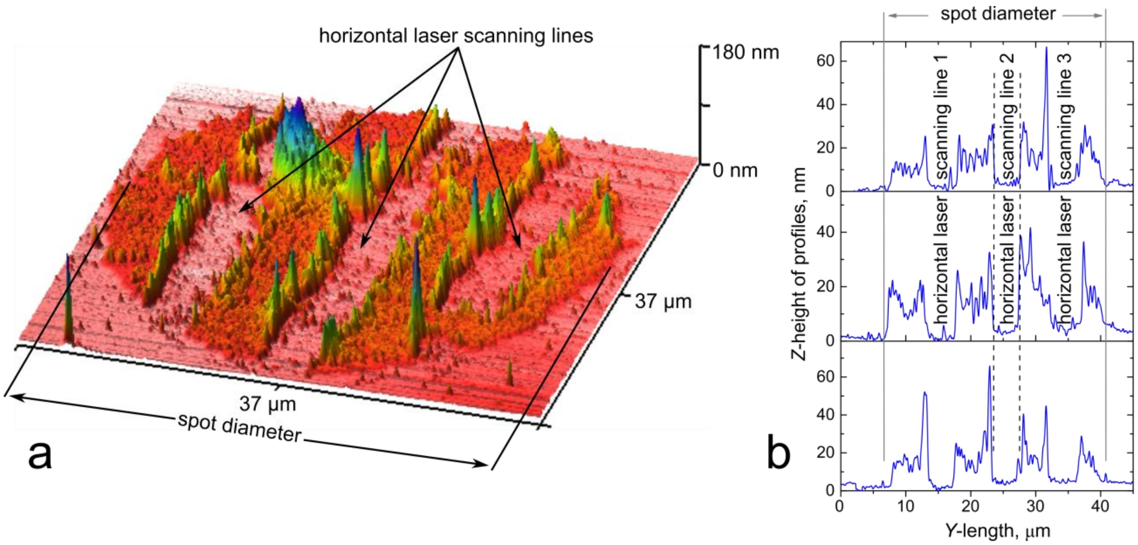

Fig. S4. (a) AFM topography of a laser-irradiated dried droplet of 195 pL suspension containing 100 nm PEG-coated Au NPs and (b) three height profiles extracted perpendicularly to laser scans
